# Assessment and histological analysis of the IPRL technique for sequential *in situ *liver biopsy

**DOI:** 10.1186/1476-5926-10-7

**Published:** 2011-08-08

**Authors:** Anthony Rowe, Lillian Zhang, Azmena Hussain, Filip Braet, Iqbal Ramzan

**Affiliations:** 1 Faculty of Pharmacy, University of Sydney, NSW 2006, Australia; 2 Australian Centre for Microscopy & Microanalysis, University of Sydney, NSW 2006, Australia

## Abstract

**Background:**

The isolated perfused rat liver (IPRL) is a technique used in a wide range of liver studies. Typically livers are assessed at treatment end point. Techniques have been described to biopsy liver in the live rat and post-hepatectomy.

**Results:**

This paper describes a technique for obtaining two full and one partial lobe biopsies from the liver *in situ *during an IPRL experiment. Our approach of retaining the liver *in situ *assists in minimising liver capsule damage, and consequent leakage of perfusate, maintains the normal anatomical position of the liver during perfusion and helps to keep the liver warm and moist. Histological results from sequential lobe biopsies in control perfusions show that cytoplasmic vacuolation of hepatocytes is a sign of liver deterioration, and when it occurs it commences as a diffuse pattern which tends to develop a circumscribed, centrilobular pattern as perfusion progresses.

**Conclusions:**

Liver lobe biopsies obtained using this method can be used to study temporal effects of drug treatments and are suitable for light and electron microscopy, and biochemical analyses.

## Background

The isolated perfused rat liver (IPRL) is a well characterised model which is commonly used to study the biology and pathobiology of the liver in various experimental settings [1-3]. IPRL has a wide range of applications, including ischemia-reperfusion [4], biochemistry [5], pharmacology [6] and immunology [7]. Previous and ongoing studies in our laboratory have used this model to examine the hepatotoxicity of kava [8].

Liver lobe biopsies during IPRL enable temporal profiles of treatments to be observed in each liver. Lobe biopsy techniques have been described using microsurgical techniques in live rats [9,10], and in perfused rat livers post hepatectomy [11]. However, detailed written and pictorial instructions for taking *in situ*, post mortem lobe biopsies are lacking. Here we describe an uncomplicated technique for obtaining two full and one partial liver lobe biopsy from liver *in situ *during an IPRL experiment, and corresponding control histological results. The histological architecture of the rat liver under these conditions is also discussed.

## Results

### Liver lobe biopsy

The liver of the anaesthetised rat is isolated and perfused as described in methods to complete a circuit with inflow via the portal hepatic vein and outflow via the suprahepatic inferior vena cava [1-3]. To avoid damaging the liver capsule, it is preferable to use fingers, moist cotton buds or blunt, plastic instruments to manipulate the liver lobes instead of sharp or toothed metal instruments. The liver should be continuously moistened with warm saline to prevent desiccation. The medial and left lateral lobes are folded cranially once creased parafilm (Pechiney Plastic Packaging Company, Chicago, IL, USA) is placed over the edge of the cut ribs to prevent puncturing of the parietal surface of these lobes.

**Figure 1:**
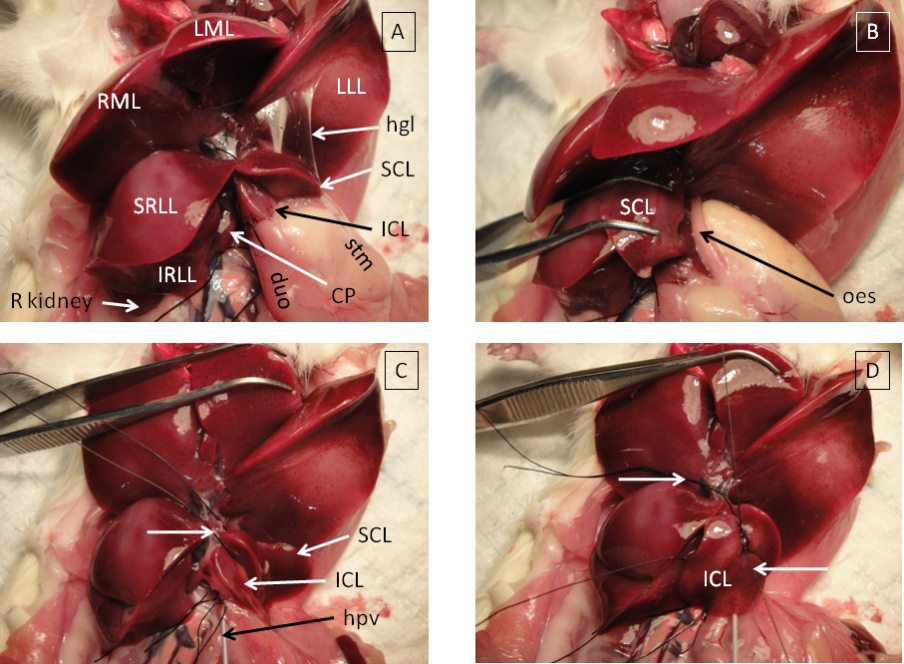
**Sequential lobe biopsy during IPRL (part I)**. This figure was prepared with a non-perfused rat liver to aid manipulation and photography. Perfused liver becomes pale brown with exsanguination. CP = caudate process, duo = duodenum, hgl = hepatogastric ligament, hpv = catheter in hepatic portal vein, ICL = inferior caudate lobe, IRLL = inferior right lateral lobe, IVC = inferior vena cava, LLL = left lateral lobe, LML = left median/middle lobe, oes = oesophagus, R kidney = right kidney, RML = right median/middle lobe, SCL = superior caudate lobe, SRLL = superior right lateral lobe, stm = stomach. A. Anatomy of the rat liver. B. Stomach and oesophagus separate SCL and ICL. C. Untied ligature placed around pedicle of SCL. D. Arrow pointing right = untied ligature around pedicle of SCL. Arrow pointing left = tied ligature around pedicle of ICL.

**Figure 2:**
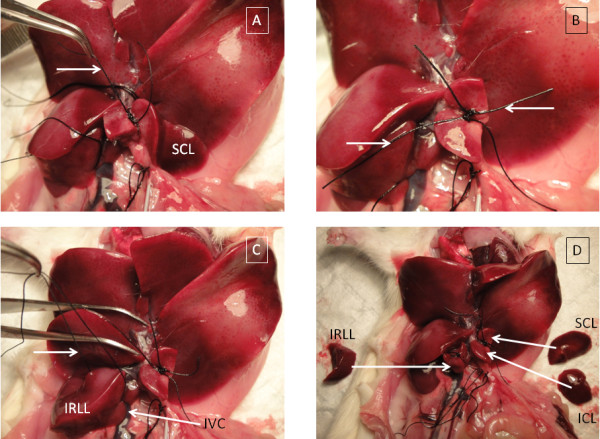
**Sequential lobe biopsy during IPRL (part II)**. A. Arrow pointing right = tied ligature around pedicle of ICL. ICL has been removed. B. Arrow pointing right = tied ligature around pedicle of ICL. Arrow pointing left = tied ligature around pedicle of SCL. Both caudate lobes have been removed. C. Arrow pointing right = untied ligature placed around body of IRLL. D. Biopsied liver lobes.

The regional anatomy of the liver is labelled (Figure 1A) according to published nomenclature [12]. The superior caudate lobe (SCL) is reflected medially to expose and section the oesophagus (Figure 1B). The stomach and spleen can then be carefully dissected away from the caudate lobes by cutting through the thin layers of peritoneum known as the hepatoduodenal and hepatogastric ligaments. A loop of 4/0 silk is placed around the pedicle of the superior caudate lobe and left untied (Figure 1C). This must be carefully fed around the pedicle rather than pulled, to prevent shearing of the liver parenchyma. A loop of 4/0 silk is similarly placed around the pedicle of the inferior caudate lobe (ICL) which is tied (Figure 1D), then this lobe is excised with scissors (Figure 2A). Once a lobe biopsy is complete, it is important to return the remaining lobes of the liver to their normal anatomical positions to allow optimum perfusion. The liver should be covered in parafilm and moistened with warm saline to prevent desiccation. The perfusion should be performed with 37°C perfusate in a temperature controlled hood.

At appropriate time points, the left lateral and medial lobes are folded cranially again, and the superior caudate lobe (Figure 2B) and the inferior right lateral lobe (IRLL) (Figure 2C) may be removed. A partial biopsy is taken of the IRLL to avoid damage to the underlying inferior vena cava. This ligature is only tied to compress the remaining liver lobe. If it is tied completely, it will cut through the lobe, resulting in leakage of perfusate. For this reason, the IRLL is the final biopsy taken at the conclusion of the IPRL experiment. If the liver is required for electron microscopy, it can then be immediately perfused with glutaraldehyde [13].

Each biopsied lobe (Figure 2D) was cut into thirds longitudinally, which were weighed and recorded. The central third was typically used for histology, and if required, the lateral thirds can be homogenised for biochemical assays.

For the duration of each IPRL experiment, the liver was even in colour, had sharply defined edges on the lobes and the perfusate was pale yellow and clear. The final transaminase levels measured in perfusate were similar to those measured in baseline serum prior to the commencement of IPRL. Bile flow reduces during perfusion (data not shown).

### Histology

**Figure 3:**
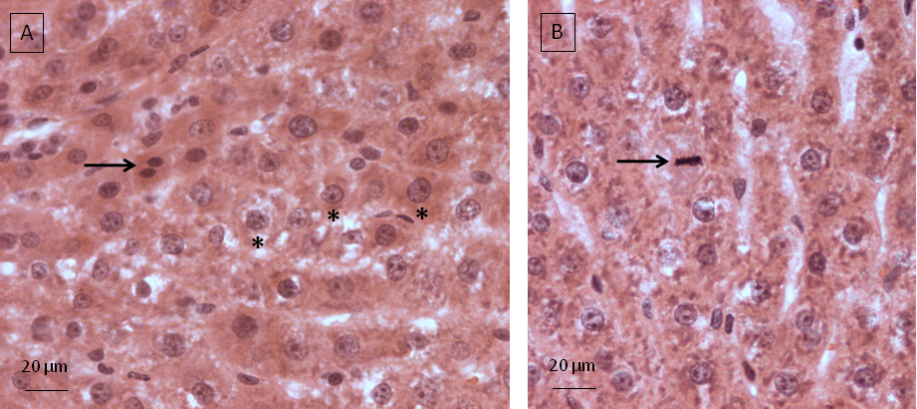
**Normal histological section of ICL**. A. Typical clear, pale staining, hepatocyte nuclei with one to two nucleoli and clumped chromatin (*). Black arrow shows a binucleate cell. B. Black arrow shows a mitotic figure.

**Figure 4:**
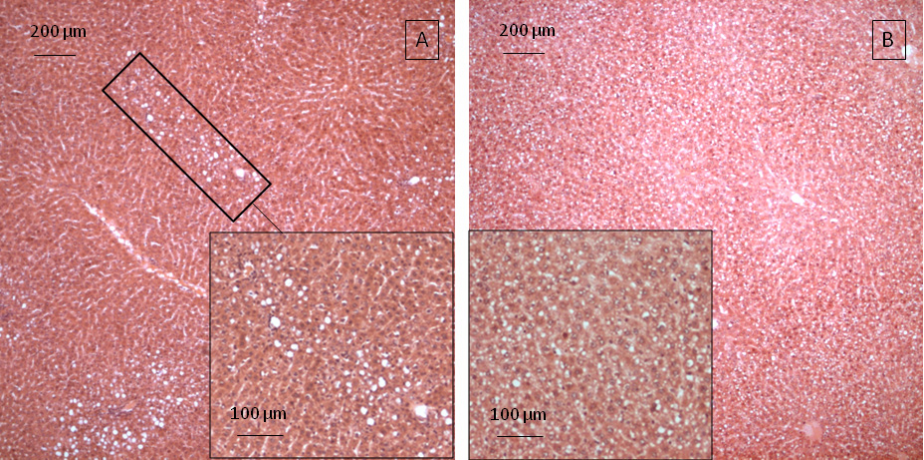
**Histological section of ICL showing vacuolation (insets show higher magnification)**. A. Mild, isolated vacuolation (black boxes). B. Severe, extensive, cytoplasmic vacuolation.

The hepatocytes in most sections of the ICL contain clear, pale staining nuclei with one to two nucleoli and clumped chromatin (Figure 3A). Occasional binucleate cells (Figure 3A) and mitotic figures (Figure 3B) are present. The cytoplasm of most hepatocytes is pale and eosinophilic with finely granular basophilic inclusions. The hepatic sinusoids and central veins are predominantly clear of erythrocytes. Fifteen out of eighteen sections taken contained either no vacuolation or diffuse pockets of mild to moderate vacuolation (Figure 4A). Sections from three out of eighteen separate ICL biopsies contained severe, extensive, cytoplasmic vacuolation (Figure 4B).

**Figure 5:**
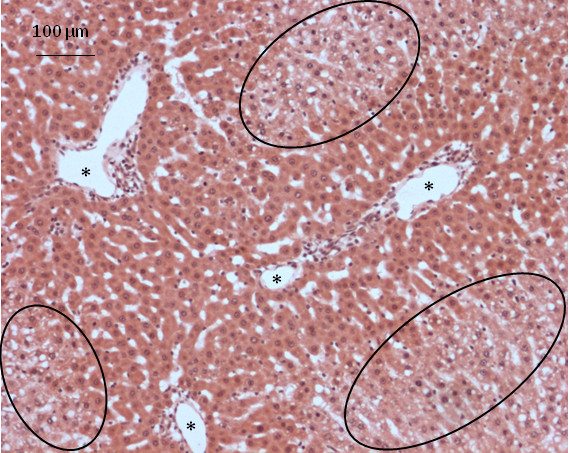
**SCL biopsy from same liver as ICL biopsy in Figure 3B**. Dilated portal triads (*) and circumscribed areas of centrilobular vacuolation (black circles).

The SCL and IRLL biopsies showed increased dilation of sinusoids, portal veins and central veins (Figure 5). Where present, areas of cytoplasmic vacuolation in these biopsies tended to become more circumscribed (Figure 5). The extent of vacuolation in the baseline ICL biopsy was indicative of vacuolisation in SCL and IRLL biopsies.

## Discussion

The technique described enables the collection of up to three biopsies of liver to be obtained during an IPRL experiment, thus providing time points for comparison of treatment effects. The ICL represents a histological baseline for the condition of the liver post-flushing. Degenerative changes seen in SCL and IRLL biopsies during control perfusions can be used to distinguish from treatment effects in non-control perfusions.

When the liver remains *in situ *during perfusion, it minimises liver capsule damage and consequent leakage of perfusate, it maintains the normal anatomical position of the liver during perfusion and it assists in keeping the liver warm and moist. Maintaining the normal anatomical position and hence circulation minimises hepatic congestion and oedema, which can be observed during perfusion as swelling of misplaced lobes.

It is important to avoid damage to the hepatic capsule as this can lead to leakage of perfusate. If sufficient leakage of perfusate occurs during an IPRL experiment, the perfusate must be replenished. When the perfusate contains a chemical or drug as treatment, the addition of fresh perfusate could be a confounding factor because it may change the ratio of the chemical or drug to metabolite present at the same time point in a non-leaking perfusion experiment.

Since the purpose of this manuscript is to provide detailed written and pictorial instructions for taking *in situ*, post mortem, lobe biopsies, the scope does not include comparisons with other techniques such as *ex-situ *isolated perfused rat liver [11] with various method variations [1,3], isolated dual perfused rat liver (an *in vitro *reperfusion model using both portal vein and hepatic artery) [14], and microsurgical techniques in live rats [9,10].

Describing patterns of histological change observed requires a clear interpretation of the arrangement of the rat liver, yet this is controversial because there are conflicting definitions of the structural/functional liver unit. These include the liver lobule (a polygon with portal triads on the exterior surrounding a central vein), the portal lobule (a triangle with central veins at each tip surrounding a portal triad) and Rappaport's liver acinus (adjacent triangular acini share a common base and comprise a diamond with central veins at the tips of the long axis and portal triads at the tips of the short axis. Adjacent acini extend into adjacent liver lobules) [15]. Acini are traditionally divided into elliptical zones extending from the short axis according to the proximity to the portal blood supply: *i.e*., zone one is periportal; zone three is pericentral; and, zone two is in between [16].

Three-dimensional studies of the angioarchitecture of the rat liver favour primary units similar to the polygonal liver lobules of human and pig liver, but without the surrounding connective tissue septum observable in pig lobules nor the septal branches of portal veins that are present in pig and human lobules [17]. These primary units are arranged into cone-shaped secondary units which drain into a common central venular tree. Histochemical studies support these findings [18,19].

Whilst the acinus is a widely used description in liver histology, the central axis of the blood supply is the terminal afferent portal venules in the vascular septum extending between portal triads. The sparsity of these septal branches in the rat makes the concept of the acinus unlikely in this species. Although the vasculature necessary to define the acinus is lacking, spheres of enzymic zonation can be defined with markers for the periportal enzyme carbamoylphosphate synthetase and the pericentral enzyme glutamine synthetase, which are consistent with the liver lobules described by three-dimensional, angioarchitectural studies [20].

Studies using dye injections into portal and hepatic veins of rat liver suggest that the structural/functional unit of the rat liver is the portal lobule [21]. The difficulty with this model is that according to angioarchitectural studies, a considerably larger portion of the blood supply to rat liver sinusoids originates from the portal venous branch. This makes it unlikely that a larger number of central veins are present to drain blood from a smaller number of portal veins, as would be the case in the triangular portal lobule design.

Using the concept of the liver lobule to describe the two dimensional histology of the rat liver, vacuolation in SCL and IRLL biopsies from control perfused livers showed a centrilobular distribution. The severe, extensive, cytoplasmic vacuolation seen in sections from three out of eighteen separate ICL biopsies may be a result of insufficient oxygenation. Vacuolation is observed in non-perfused livers anywhere from 30 seconds to 30 minutes post-mortem [22]. Anoxia causes an increase in hepatocyte permeability and high intrahepatic pressure following death forces sinusoidal plasma into the hepatocytes. Alternatively, fluctuations in pressure during IPRL may have a similar effect. This may occur either with or without anoxia, particularly using a constant flow rate setup. Since most sections display predominantly open sinusoids which are clear of plasma and blood cells, and open bile canaliculi in the periportal areas, tissues obtained from these biopsies make suitable specimens for use in electron microscopy [13].

## Conclusions

This is a technique for obtaining serial lobe biopsies from an IPRL whilst *in situ*, which minimises damage to the hepatic capsule during preparation and enables temporal aspects of treatments to be observed. Lobe biopsies obtained are suitable for light and electron microscopy, and biochemical analyses. The main degenerative change observed with light microscopy in control IPRL is cytoplasmic vacuolation. This is usually mild with a centrilobular distribution.

## Methods

### Isolated Perfused Rat Liver (IPRL)

These studies were approved by the Animal Ethics Committee of The University of Sydney. The IPRL procedure was performed as described previously [23]. After a midline incision, 1 ml blood was collected from the caudal vena cava for serum transaminase measurements, and then 500 IU heparin in 0.5 ml (Pfizer, West Ryde, NSW, Australia) was injected. Liver perfusion was commenced with non-recirculating, lactated Ringer's solution (compound sodium lactate = Hartmann's solution - Baxter, Old Toongabbie, NSW, Australia) until the first lobe biopsy (ICL) was obtained. This was performed by infusion from sterile bags manufactured for intravenous fluid therapy and had no additional oxygenation. Once the ICL biopsy was obtained, the perfusate was switched to 100 ml acellular, recirculating Krebs-Henseleit buffer. The composition of the buffer was as follows: 118 mM NaCl, 25 mM NaCO_3_, 4.7 mM KCl, 2.5 mM CaCl_2_.2H_2_O, 1.3 mM NaH_2_PO_4_.2H_2_O, 1.2 mM MgSO_4_.7H_2_O, 2% bovine serum albumin (BSA, fraction V, Sigma, Sydney, Australia) and 0.2% glucose [2]. Acellular perfusate is commonly used in IPRL experiments and avoids additional complications and variables associated with blood components [24-28]. This was continuously mixed in a reservoir on a magnetic stirrer and aerated with Carbogen (95% O_2 _+ 5% CO_2_), which was bubbled into the reservoir rather than using an oxygenator to avoid kavalactone adsorption onto oxygen permeable tubing. This solution was recirculated at a constant flow of 16 ml/min using a peristaltic pump (MasterFlex, Cole-Parmer Instrument Company, Chicago, IL). To support bile flow, 60 mM taurocholic acid (Sigma, Castle Hill, NSW, Australia) in Krebs-Henseleit buffer was pumped into the perfusate reservoir at 1 ml h^-1 ^using a syringe infusion pump (Harvard Apparatus, Holliston, MA). Liver viability was judged on the basis of gross appearance, histology, liver transaminases and bile flow.

### Liver histology

All reagents used for histopathology processing were Fronine brand (Lomb Scientific, Taren Point, NSW, Australia). Liver lobe biopsies were fixed by overnight immersion in 10% neutral-buffered formalin. Tissues were then placed in embedding cassettes (ProSciTech, Thuringowa Queensland, Australia) dehydrated through graded ethanol, cleared in xylene and infiltrated with paraffin wax in an Excelsior ES Tissue Processor (Thermo Fisher Scientific Australia, Scoresby, Victoria, Australia). Processed tissues were embedded in paraffin using a Shandon Histocentre 3 (Thermo). Five micron tissue sections were cut using a Leica RM2235 manual rotary microtome (North Ryde, NSW, Australia), stained with haematoxylin and eosin, and mounted on glass slides. Images were obtained using a Nikon Eclipse E800 fluorescence microscope (Nikon, Lidcombe, NSW, Australia) equipped with a PCO SensiCam 12-bit colour CCD camera.

## Competing interests

The authors declare that they have no competing interests.

## Authors' contributions

AR developed the method, obtained histology images and drafted the manuscript. LZ and AH assisted with method development and collection of images. FB and IR assisted in the preparation of the manuscript. All authors read and approved the final manuscript.
